# Hypoxia induced‐disruption of lncRNA TUG1/PRC2 interaction impairs human trophoblast invasion through epigenetically activating Nodal/ALK7 signalling

**DOI:** 10.1111/jcmm.17450

**Published:** 2022-06-21

**Authors:** Mengsi Hu, Yao Wang, Yanping Meng, Jinxiu Hu, Jiao Qiao, Junhui Zhen, Decai Liang, Minghua Fan

**Affiliations:** ^1^ Department of Nephrology, Shandong Provincial Hospital, Cheeloo College of Medicine Shandong University Jinan China; ^2^ Department of Nephrology Shandong Provincial Hospital Affiliated to Shandong First Medical University Jinan China; ^3^ Department of Obstetrics and Gynecology, The Second Hospital, Cheeloo College of Medicine Shandong University Jinan China; ^4^ Department of Pathology, Qilu Hospital, Cheeloo College of Medicine Shandong University Jinan China; ^5^ School of Statistics and Data Science, LPMC and KLMDASR Nankai University Tianjin China

**Keywords:** H3K27me3, hypoxia, nodal signalling, placenta, PRC2, preeclampsia, trophoblast invasion, TUG1

## Abstract

Inadequate trophoblastic invasion is considered as one of hallmarks of preeclampsia (PE), which is characterized by newly onset of hypertension (>140/90 mmHg) and proteinuria (>300 mg in a 24‐h urine) after 20 weeks of gestation. Accumulating evidence has indicated that long noncoding RNAs are aberrantly expressed in PE, whereas detailed mechanisms are unknown. In the present study, we showed that lncRNA Taurine upregulated 1 (TUG1) were downregulated in preeclamptic placenta and in HTR8/SVneo cells under hypoxic conditions, together with reduced enhancer of zeste homolog2 (EZH2) and embryonic ectoderm development (EED) expression, major components of polycomb repressive complex 2 (PRC2), as well as activation of Nodal/ALK7 signalling pathway. Mechanistically, we found that TUG1 bound to PRC2 (EZH2/EED) in HTR8/SVneo cells and weakened TUG1/PRC2 interplay was correlated with upregulation of Nodal expression via decreasing H3K27me3 mark at the promoter region of Nodal gene under hypoxic conditions. And activation of Nodal signalling prohibited trophoblast invasion via reducing MMP2 levels. Overexpression of TUG1 or EZH2 significantly attenuated hypoxia‐induced reduction of trophoblastic invasiveness via negative modulating Nodal/ALK7 signalling and rescuing expression of its downstream target MMP2. These investigations might provide some evidence for novel mechanisms responsible for inadequate trophoblastic invasion and might shed some light on identifying future therapeutic targets for PE.

## 1 INTRODUCTION

Preeclampsia (PE) is a major cause of maternal death worldwide, characterized by newly onset of hypertension (>140/90 mmHg) and proteinuria (>300 mg in a 24‐h urine) after 20 weeks of gestation.^1^, ^2^ Successful placental and foetal development during the progression of pregnancy require adequate invasion of extravillous trophoblast cells into the uterine, and impaired trophoblastic invasion and spiral artery remodelling are widely considered as hallmarks of PE.^3^, ^4^ According to the literature, reduced trophoblastic invasion and spiral artery remodelling may be due to abnormal trophoblast differentiation, migration, angiogenesis and apoptosis, which may cause reduced uteroplacental perfusion and trigger hypoxia and inflammatory responses in the placenta, and may finally result in the occurrence of PE.^5^, ^6^, ^7^, ^8^, ^9^, ^10^ Besides, a variety of other potential molecular mechanisms have been suggested, including oxidative stress, endoplasmic stress, altered NK cell signalling and others.^11^ However, over the decades, a full understanding of the pathogenesis of PE remains elusive.

Accumulating evidence has indicated that genome‐wide long noncoding RNAs (lncRNAs) expression patterns are aberrantly expressed in preeclamptic placenta.^12^ LncRNAs are non‐protein‐coding transcripts longer than 200 nucleotides and have been implicated to play critical roles in various biological processes.^13^ Many lncRNAs have emerged as crucial molecular regulators in trophoblastic and placental dysfunction, and thereby involved in the development of PE, potentially through regulating the migration and invasion of trophoblast cells, inhibiting trophoblast proliferation and cell cycle, regulating angiogenesis and decidualization, and participating in inflammation and immune regulation.^14^, ^15^, ^16^, ^17^ Taurine upregulated 1 (TUG1) is a 7598‐bp lncRNA gene located on human chromosome 22q12.2, characterized as an oncogene closely correlated with cell proliferation, invasion, migration, metastasis and apoptosis.^18^ TUG1 upregulates SIRT1, which further leads to the activation of Wnt/β‐catenin signalling pathway, and promotes proliferation and invasion of cervical cancer cells.^19^ Over‐expressed TUG1 drives proliferation and migration of pancreatic cancer via epithelial‐mesenchymal transition pathways including TGF‐β‐Smad signalling.^20^ Of interest, both Wnt/β‐catenin and TGF‐β‐Smad signalling pathways have been proposed to be dysregulated in preeclamptic placentas,^11^, ^21^ suggesting that TUG1 might be playing a role in PE. Recently, studies showed that TUG1 was significantly reduced in PE placental tissue and could prohibit the migration and invasion of trophoblast‐like cells in a TUG1/microRNA (miR)‐204‐5p axis‐dependent manner.^22^ Other studies demonstrated that TUG1 expression was downregulated by hypoxia‐induced miR‐218 through targeting FOXP1 and TUG1 might act as an essential determinant of spiral artery remodelling impairment in PE.^23^ Nevertheless, the involvement of TUG1 in PE and trophoblast invasion is still poorly understood.

Many lncRNAs bind to chromatin‐modifying proteins and recruit their catalytic activity to specific sites in the genome, suggesting that lncRNAs may function as important epigenetic modulators.^24^ Epigenetic mechanisms that regulate histone methylation with dysregulation of lncRNAs have been identified in the development of PE.^25^ Polycomb repressive complex 2 (PRC2) is a methyltransferase, which contains four core components (in humans): enhancer of zeste homolog2 (EZH2), embryonic ectoderm development (EED), suppressor of zeste 12 protein homologue (SUZ12) and RbAp46/48. In the prevailing model, PRC2 is recruited to specific genomic locations where it trimethylates H3K27 to repress transcription of specific genes.^26^, ^27^, ^28^ In prostate cancer cells, EZH2 expression can be targeted by miR‐101 upon hypoxia‐inducible factor 1α/β (HIF‐1α/β) induction.^29^ HIF is a transcription factor protected from degradation in various cells under hypoxic conditions and plays an essential role in modulating responses to hypoxia by controlling the expression of multiple genes.^10^, ^30^ HIF‐1α is stabilized when the concentrations of oxygen are below the specific critical oxygen threshold thus accumulating in the hypoxic cell, and HIF‐1β is constitutively present in the cell nucleus.^31^ Numerous studies have illustrated that hypoxia‐reperfusion‐type injuries to the placenta in PE is due to inadequate placental perfusion as a result of insufficient trophoblast invasion and spiral artery remodelling; and HIF‐1α and its targets genes, including vascular endothelial growth factor (VEGF), VEGF‐receptor 1, placental growth factor, platelet‐derived growth factor and matrix metalloproteinases (MMPs), are altered in preeclamptic placenta and thus may be playing a pivotal role during the development of PE.^10^, ^32^ Nonetheless, it is unknown how the key component of PRC2, EZH2 to be specific, would be affected in trophoblast under hypoxic conditions and in the pathogenesis of PE.

Reportedly, EZH2 cooperates with Smad3 during dedifferentiation of neuroretinal epithelial cells in response to TGF‐β, and regulates cellular plasticity.^33^ Nodal is a member of the TGF‐β superfamily, which signals through type II receptors (activin receptors IIA and IIB) and I receptors (activin receptor‐like kinases [ALK] 4 and 7).^34^ In the pathogenesis of PE, Nodal inhibits trophoblast migration and invasion through activating ALK7.^34^ Studies have shown that JMJD3 is recruited to promoters of the Nodal gene in mouse embryonic stem cells (ESCs), leading to removal of the repressive H3K27me3 mark and thereby initiating mesendoderm differentiation.^35^, ^36^ Nonetheless, the interactions between PRC2 and Nodal signalling in PE are not well studied. Of note, recent studies have demonstrated a significant reduction of TUG1 levels in placenta samples of women with PE, which might contribute to PE development with epigenetic regulation of RND3 through binding to EZH2.^37^ Thus, it is of interest to further identify the interplay among TUG1, PRC2 and Nodal signalling during the development of PE.

Collectively, in the present study, we seek to investigate the role of Nodal signalling, the interactions between TUG1 and PRC2, and how these interactions might affect Nodal signalling in trophoblast dysfunction under hypoxic conditions, thus uncovering a potential molecular mechanism for the pathogenesis of PE and identifying future therapeutic targets.

## 2 MATERIALS AND METHODS

### 2.1 Placental samples and patients

A total of 108 patients were recruited from the Department of Obstetrics and Gynecology of The Second Hospital of Shandong University from 2017 to 2019. Placental tissues were collected immediately after caesarean deliveries, snap‐frozen with liquid nitrogen and stored at −80°C before processing. Pregnant patients with chronic renal disease, hypertension before pregnancy, placental abruption or placenta praevia, anaemia or other haematological disease, gestational diabetes, or foetal distress syndrome was excluded in the present study. Patients' clinical characteristics were recorded in Table 1. The study was approved by the Clinical Research Ethics Committee of The Second Hospital of Shandong University and all informed consents were obtained from participants according to the Declaration of Helsinki (Ethical approval code KYLL‐2017 [GJ]P‐0019).

**TABLE 1:** Clinical characteristics of normal and preeclamptic pregnancies

Variables	NC (*n* = 54)	PE (*n* = 54)	*p* value
Maternal age (year)	29.81 ± 4.599	31.52 ± 4.379	0.053
Maternal weight (kg)	69.25 ± 8.187	70.52 ± 9.077	0.412
Systolic blood pressure (mmHg)	115.02 ± 9.614	162.65 ± 15.847	<0.01
Diastolic blood pressure (mmHg)	72.747 ± 6.894	108.44 ± 10.621	<0.01
Proteinuria (g/24 h)	0.17 ± 0.069	2.72 ± 1.864	<0.01
Infant body weight (g)	3409.61 ± 477.831	2219.02 ± 786.723	<0.01

### 2.2 Cell culture

The human immortalized first‐trimester trophoblast cell line HTR‐8/SVneo cells were cultured in RPMI‐1640 media supplemented with 10% foetal bovine serum (FBS; HyClone) and 100 U/ml penicillin and streptomycin at 37°C in 5% CO_2_ (Gibco BRL/Invitrogen). For incubations in normoxic conditions, cells were cultured in a CO_2_ incubator supplied with 20% O_2_, 5% CO_2_ and 75% N_2_. For incubations in hypoxia, cells were placed in a chamber filled with 1% O_2_, 5% CO_2_ and 94% N_2_ for 24 h. Oxygen concentration was monitored using an oxygen electrode microrespirometer (OS1000; Oxygen Sensors, Inc). At the end of the incubation, cells were collected for different analysis.

### 2.3 RNA interference

Plasmid vectors that target human EZH2 (5′‐GCTAGGTTAATTGGGACCAAA‐3′; EZH2 shRNA), EED (5′‐CCAGAGACATACATAGGAATT‐3′; EED shRNA), TUG1 (5′‐CTGTTGACCTTGCTGTGAGAA‐3′; TUG1 shRNA), Nodal (5′‐GCAGAACTGGACGTTTGCTTT‐3′; Nodal shRNA), ALK7 (5′‐CGGAGGAATTGTTGAGGAGTA‐3′; ALK7 shRNA), expression vectors for EZH2, EED, TUG1, empty control vectors and a scrambled shRNA (5′‐GACTTCATAAGGCGCATGC‐3′) were purchased from Cyagen Biosciences Inc. Transfection of HTR8/SVneo cells was performed (1 × 10^6^ cells/well in a six‐well plate) with the indicated plasmids using Lipofectamine 3000 reagent (Invitrogen, Life Technologies Corporation). Western blot analysis and real‐time reverse transcriptase PCR were used to validate the efficiency of shRNAs and expressing vectors. After a 24‐h incubation with shRNAs or expression vectors, as well as control vectors, HTR8/SVneo cells were then further cultured under hypoxic conditions for another 24 h.

### 2.4 RNA extraction and quantitative real‐time PCR

Real‐time reverse transcriptase‐PCR was used to detect the gene expression of placental tissues or HTR‐8/SVneo cells. Extraction and concentration calculation of total RNA were according to the manufacturer instructions. Aliquots of total RNA (1.0 μg each) from each sample were reverse transcribed into cDNA according to instructions of PrimeScript “RT Reagent Kit” (Takara). Briefly, after reverse transcription of total RNA, cDNA was used as a template for the PCR reactions using specific primer pairs for TUG1 gene (sense, 5′‐TAGCATCTCACAAGGCTGCAC‐3′; antisense, 5′‐TCGGTCACAAAATGCATAGAGGT‐3′), TUG1 gene (for RNA immunoprecipitation (RIP); sense, 5′‐GCCTTGTGCCGAGAGATGTT‐3′; antisense, 5′‐ATCGGAAATGCCCAGCAAGT‐3′) and Nodal gene (for ChIP; sense, 5′‐CTGTCTCAGCATCAAGGCGT‐3′; antisense, 5′‐GGGATCTAGGGCTCCTGGTTT‐3′). Amplification was performed using SYBR “Premix Ex TaqTM Kit (Takara) in the LightCycler” 480 Real‐Time PCR system (Roche Applied Science, F. Hoffmann‐La Roche Ltd). The primers were purchased from Sangon Biotech Co., Ltd.

### 2.5 Western blot analysis

The total protein was extracted from placental tissues and HTR‐8/SVneo cells with ice‐cold lysis buffer containing proteinase inhibitors and phosphatase inhibitors. About 50 μg of total protein was separated by SDS‐PAGE and transferred to polyvinylidene fluoride membranes; the membranes were then blocked with 5% milk or BSA for 1 h and incubated at 4°C overnight with primary antibodies against the following target proteins: EZH2 (1:1000), EED (1:1000), Nodal (1:1500), ALK7 (1:1000), H3K27me3 (1:1000), MMP2 (1:1000), H3 (1:1000) and β‐actin (1:1000). The membranes were then washed three times with TBST for 5 min. and incubated with species‐specific peroxidase‐conjugated secondary antibodies diluted in blocking buffer for 1 h at room temperature. Specific bands were detected using the ECL system and the Bio‐Rad electrophoresis image analyzer (Bio‐Rad).

### 2.6 Immunohistochemistry

Briefly, placenta sections were immunostained with primary H3K27me3 (1:500) antibodies at 4°C overnight and HRP‐labelled goat anti‐rabbit IgG the next day. Staining was completed by incubation with diaminobenzidine chromogen solution (DAB; Solarbio). The sections were counterstained with Harris's haematoxylin (Solarbio), and then dehydrated and mounted. Images were captured on an inverted phase microscope (Leica Microsystems GmbH).

### 2.7 Chromatin immunoprecipitation assay (ChIP)

According to the EZ‐Magna CHIP™ HiSens kit (#17‐10461; Millipore), 1 × 10^7^ HTR‐8/SVneo cells from different treatments were fixed with 1% paraformaldehyde, quenched with glycine, washed with cold PBS and re‐suspended in nuclei isolation buffer with a protease inhibitor cocktail. The chromatin was sonicated on wet ice to obtain chromatin fragment lengths between 200 and 1200 bp. The protein‐DNA complexes were incubated with 1 μg of anti‐H3K27me3, or non‐specific IgG antibodies couple with protein A/G beads overnight followed by washing and elution. Cross‐links were reversed at 65°C for 2 h and 95°C for 15 min. DNA was purified and subjected (20 ng each) to real‐time PCR analysis.

### 2.8 RNA‐binding protein immunoprecipitation (RIP)

RNA‐binding protein immunoprecipitation was performed using Magna RIP kit (#17‐10521). In brief, HTR‐8/SVneo cells from different treatments were fixed by 0.3% formaldehyde and quenched with glycine, and scraped to pellet cells, which were resuspended and collected by high‐speed vortex and spinning at 800 *g* at 4°C for 5 min. For sonication, nuclei pellets were resuspended in 500 μl RIP Cross‐linked Lysis Buffer containing protease/RNase inhibitors and sonicated on wet ice using a sonicator (SONICS&MATERALS, Inc). Antibody‐beads complex was prepared, and 50 μl sheared cross‐linked chromatin was incubated with antibody‐beads with rotation overnight at 4°C. Cross‐link reversal was done by incubating the beads in 200 μl Elution Buffer containing 10% SDS and proteinase K with shaking at 60°C for 30 min. RNA extraction was according to the manufacturer instructions. Aliquots of total RNA (150 ng each) from each sample were reverse transcribed into cDNA and the enrichment of RNA was determined by real‐time PCR.

### 2.9 Transwell invasion assay

Briefly, 50 μl of diluted 1:4 Matrigel (BD Biosciences) in serum‐free RPMI 1640 medium was added to the upper chambers of 24‐well transwell inserts (Corning Costar Corp.) and incubated at 37°C overnight to enable solidification. HTR‐8/SVneo cells treated with different conditions were harvested with trypsin and re‐suspended in serum‐free RPMI1640 medium, and the upper chambers of transwell inserts coated with Matrigel were seeded with 5 × 10^4^/ml cells, which were then allowed to attach at 37°C for 24 h, with lower compartments filled with 600 μl of RPMI 1640 medium containing 10% foetal bovine serum. Non‐invading cells were removed from the upper surface of the membrane, and invading cells were fixed with 4% paraformaldehyde and stained with haematoxylin. The number of invading cells in 10 random fields on the underside of the membrane was counted by using an inverted microscope at a magnification of 200× (Leica Microsystems GmbH).

### 2.10 Statistical analysis

Experiments were performed at least three times. Values were reported as mean ± SD. Data were analysed using SPSS 19.0 software. Statistical significance was assessed using Student's *t*‐test, one‐way anova and LSD‐*t* test. *p* < 0.05 were considered to be statistically significant.

## 3 RESULTS

### 3.1 Levels of lncRNA TUG1 and PRC2 were downregulated in preeclamptic placenta and in hypoxic trophoblast

**FIGURE 1:**
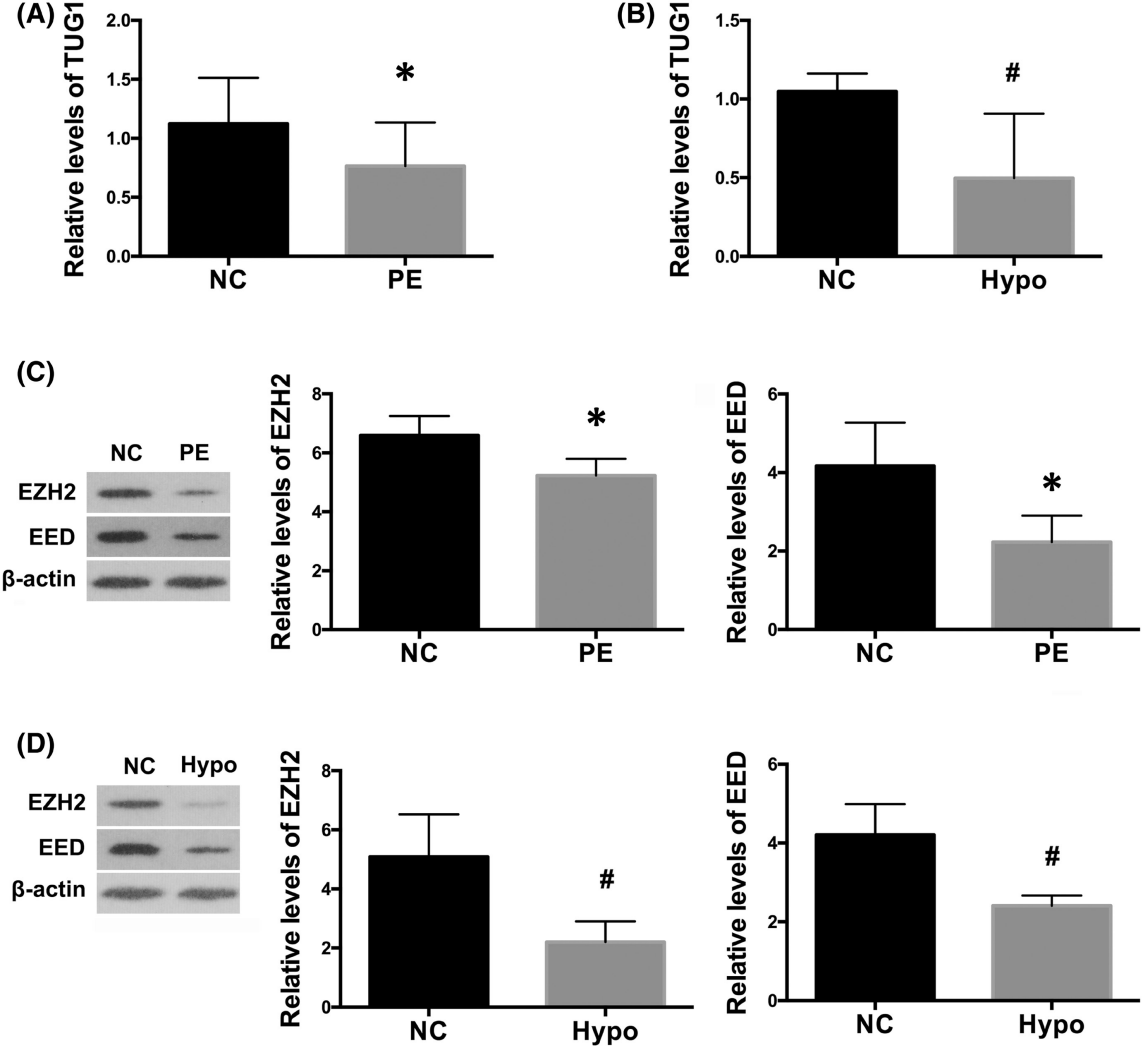
Levels of lncRNA TUG1 and PRC2 were downregulated in preeclamptic placenta and in hypoxic trophoblast. PCR analysis showed the expression levels of TUG1 in placental samples from preeclamptic patients (A) and in HTR8/SVneo cells cultured under hypoxia for 24 h (B). Values denote the mean ± SD; *p < 0.01 versus normal control (NC); ^#^ *p* < 0.05 versus NC. (C) Western blot analysis demonstrated that expression of EZH2 and EED, two major components of PRC2, were both diminished in preeclamptic placentas (C), as well as in hypoxic HTR8/SVneo cells (D). Values denote the mean ± SD; *p < 0.01 versus NC; ^#^ *p* < 0.05 versus NC

To determine expression levels of lncRNA TUG1 during normal and preeclamptic pregnancies, real‐time PCR was performed in the placental samples from a cohort of 54 pairs of severe PE patients and controls. Clinical characteristics of these normal and preeclamptic pregnancies were shown in Table 1. There were no significant differences between severe PE patients and controls in terms of maternal age or weight (*p* > 0.05). Results showed that levels of TUG1 were significantly lower in patients with severe PE, compared with controls (*p* < 0.01; Figure 1A). Previous studies have indicated that physiological angiogenesis was triggered by hypoxia during first trimester, while prolonged hypoxia would impact the expression angiogenesis‐associated growth factors, redirect placental organization, and damage invasive property of trophoblast cells and uterine spiral artery remodelling, thereafter resulted in placental dysfunction in PE.^30^, ^31^, ^38^ Thus, to further identify the role of TUG1 in trophoblast function, we cultured HTR8/SVneo cell, a normal trophoblast cell line, under hypoxic conditions (1% O_2_) for 24 h and found that levels of TUG1 were significantly lower after hypoxia treatment than cells under ambient air (*p* < 0.05; Figure 1B).

Next, we were interested to assess the expression of EZH2 and EED in PE, which are two important components of PRC2. Our results demonstrated that protein levels of EZH2 and EED were both reduced in PE placenta, as compared to normal controls (*p* < 0.01; Figure 1C). In HTR8/SVneo cells, it was shown that hypoxia led to decreased expression of EZH2 and EED, in comparison with cells under normoxic conditions (*p* < 0.05; Figure 1D). These results implicated that TUG1 and PRC2 might be of great significance during the pathogenesis of PE.

### 3.2 Hypoxia resulted in reduced H3K27me3 mark by PRC2 at the promoter of Nodal gene in trophoblast

**FIGURE 2:**
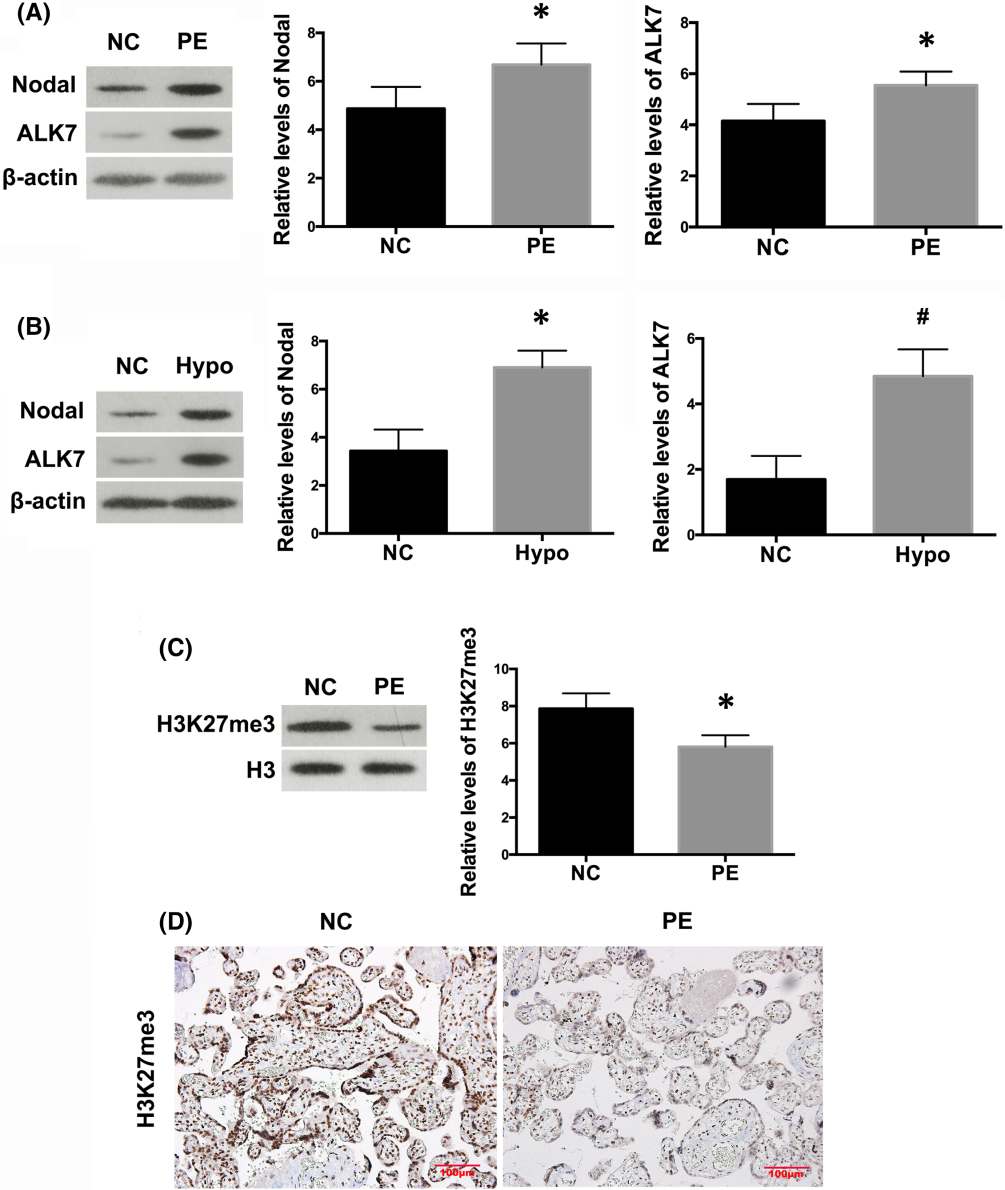
Activation of Nodal/ALK signalling pathway in preeclamptic placenta and in hypoxic trophoblast. Western blot analysis showed significantly upregulated protein levels of Nodal and ALK7 in both preeclamptic placentas (A) and HTR8/SVneo cells in hypoxia for 24 h (B), in comparison with their controls, respectively. Values denote the mean ± SD; *p < 0.01 versus NC; ^#^ *p* < 0.05 versus NC. (C) Western blot demonstrated the alterations of H3K27me3 in placental samples from PE patients, compared with normal placentas. Values denote the mean ± SD; *p < 0.01 versus NC. (D) Immunohistochemistry showed H3K27me3 was predominantly expressed in the nuclei of trophoblast cell in normal pregnancy, the staining of which was markedly decreased in preeclamptic placenta. Scale bar = 100 μm

Previous studies have demonstrated that Nodal signalling might participate in the pathogenesis of PE.^34^ In accordance with these studies, we showed that protein expression of Nodal was significantly elevated in placental samples from women with severe PE (*p* < 0.01; Figure 2A), which occurred concomitantly with enhanced expression of its downstream kinase ALK7 (*p* < 0.01; Figure 2A).

**FIGURE 3:**
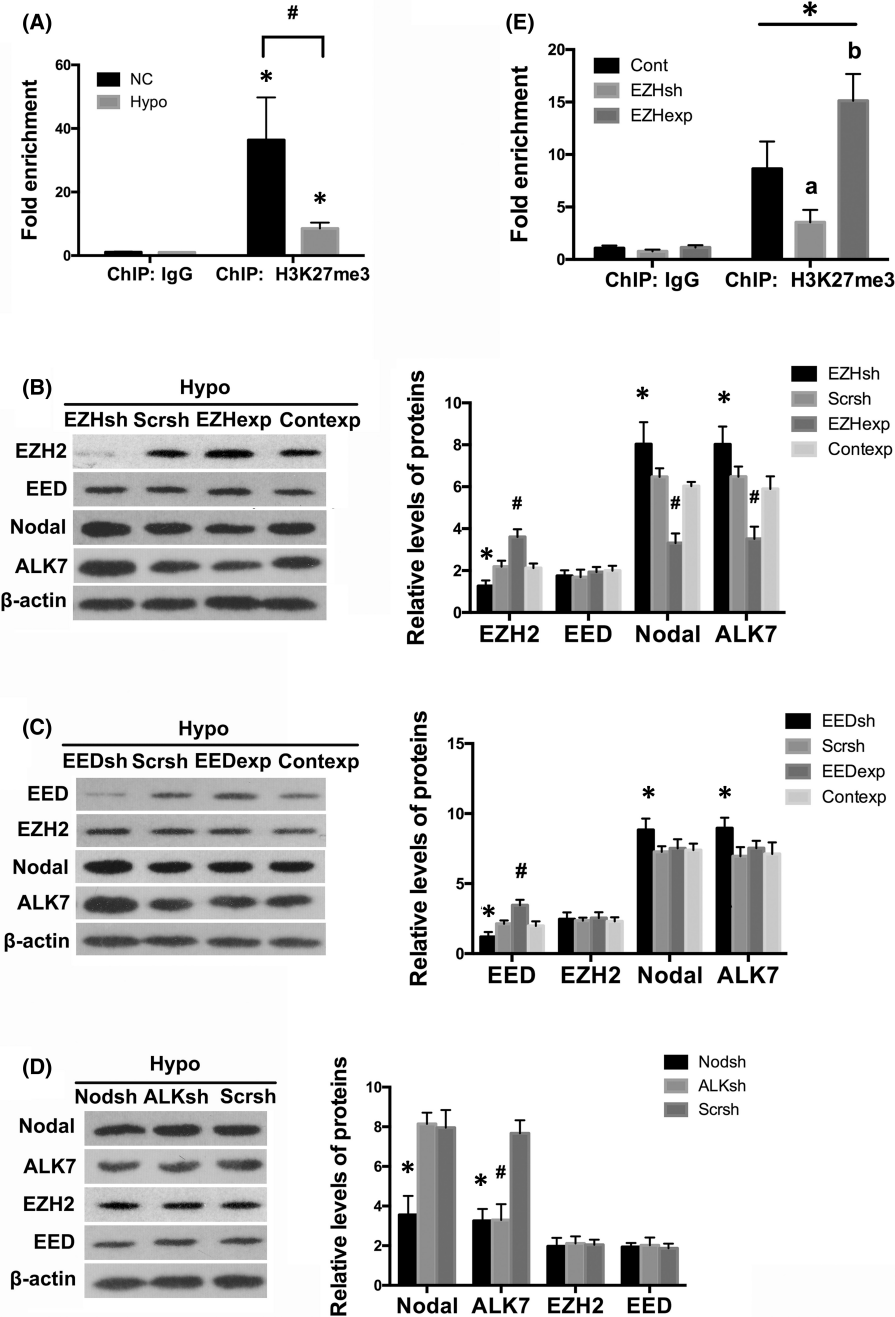
PRC2 epigenetically regulated Nodal/ALK7 signalling in trophoblast in hypoxia. (A) ChIP analysis was performed in cultured HTR8/SVneo cells with or without hypoxia treatment. Real‐time PCR was used for the detection of ChIP signals at the promoter of Nodal gene. Values denote the mean ± SD; *p < 0.01 versus IgG; ^#^ *p* < 0.01 versus NC. (B, C) Western blot analysis showed the impacts of gene interference with EZH2 or EED using either shRNAs or expressing vectors on protein expression of EZH2, EED, Nodal and ALK7 under hypoxic conditions. Values denote the mean ± SD; *p < 0.05 versus Hypoxia+ Scramble shRNA, ^*#*^ *p* < 0.05 versus Hypoxia+ Control expressing vector. (D**)** Western blot demonstrated the effects of gene knockdown of Nodal or ALK7 by shRNAs on protein expression of EZH2, EED and Nodal/ALK7 signalling pathway under hypoxia conditions. Values denote the mean ± SD; *^#^ *p* < 0.01 versus Hypoxia+ Scramble shRNA. (E) ChIP‐qPCR analysis showed differential ChIP signals at the promoter of Nodal gene in trophoblasts in hypoxia after EZH2 gene knockdown or overexpression. Values denote the mean ± SD; *p < 0.01 versus IgG, ^a^ *p* <0.05 versus Hypoxia+ Control vector, ^b^ *p* <0.01 versus Hypoxia+ Control vector

To illustrate the correlation between PRC2 and Nodal signalling, we examined the levels of Nodal and ALK7 in HTR8/SVneo cells, and found that hypoxia significantly gave rise to the expression of Nodal (*p* < 0.01) and ALK7 (*p* < 0.05; Figure 2B), in concert with downregulation of PRC2 proteins. Prevailingly, PRC2 is recruited to trimethylate H3K27 to repress specific genes.^26^, ^27^, ^28^ In the present study, we found by Western blot that levels of H3K27me3 were decreased in the placental samples of women with severe PE (*p* < 0.01; Figure 2C). Immunohistochemistry showed that in normal placenta, H3K27me3 was strongly stained and predominantly expressed in the nuclei of trophoblasts, whereas in preeclamptic placenta the staining of H3K27me3 was markedly weakened (Figure 2D). And we performed ChIP assay in HTR8/SVneo cells and found that fragments of the Nodal promoter region were amplified by H3K27me3 antibodies compared with a non‐specific IgG control under normal conditions (*p* < 0.01; Figure 3A). Stimulation with hypoxia for 24 h, however, led to markedly diminished enrichment of Nodal promotor fragments in ChIP samples, in comparison with cells under ambient air (*p* < 0.01; Figure 3A).

Next, we transfected the trophoblasts with different vectors, respectively: shRNAs for EZH2, EED, Nodal or ALK7, or expressing vectors for EZH2 or EED. Results demonstrated that EZH2 gene knockdown led to further decreased EZH2 expression (*p* < 0.05; Figure 3B), as well as further decreased H3K27me3 mark at the promoter of Nodal gene in HTR8/SVneo cells under hypoxic conditions (*p* < 0.05; Figure 3E), without interfering EED expression (*p* > 0.05; Figure 3B). Nevertheless, EZH2 knockdown resulted in a further degree of increase in both Nodal and ALK7 expression (*p* < 0.05; Figure 3B). EZH2 overexpression not only rescued EZH2 expression (*p* < 0.01; Figure 3B), but also caused a remarkable increase in trimethylation of H3K27 at the promoter of Nodal gene in trophoblasts under hypoxic conditions, in comparison with hypoxic cells with control vectors (*p* < 0.01; Figure 3E). In the meantime, EZH2 overexpression was able to partially abrogate Nodal (*p* < 0.01) and ALK7 (*p* < 0.05) overexpression resulted from hypoxia (Figure 3B), but hardly affected EED expression in hypoxia (*p* > 0.05; Figure 3B).

Silencing Nodal gene expression under hypoxic conditions led to decreased expression of Nodal (*p* < 0.01) and ALK7 (*p* < 0.01; Figure 3D), compared with hypoxic trophoblasts transfected with Scramble shRNAs. Nevertheless, Nodal knockdown did not remarkably affect expression of EZH2 or EED (*p* > 0.05; Figure 3D). ALK7 shRNAs downregulated ALK7 expression (*p* < 0.01; Figure 3D), whereas hardly impacted expression of Nodal, EZH2 or EED (*p* > 0.05; Figure 3D). These results suggested Nodal/ALK7 signalling might be a downstream pathway of PRC2 dysregulation under hypoxic conditions through an epigenetic regulating mechanism.

Of interest, under hypoxic conditions, silencing EED gene resulted in a further degree of EED downregulation (*p* < 0.05; Figure 3C), along with enhanced expression of Nodal (*p* < 0.05) and ALK7 (*p* < 0.01; Figure 3C), without interfering the expression of EZH2 (*p* > 0.05; Figure 3C). Overexpression of EED gene, although elevated EED expression under hypoxic condition (*p* < 0.05; Figure 3C), did not significantly prohibit Nodal/ALK7 signalling activation in hypoxic trophoblasts (*p* > 0.05; Figure 3C).

### 3.3 Damaged TUG1/PRC2 interaction caused activation of Nodal/ALK7 signalling in trophoblast under hypoxic conditions

**FIGURE 4:**
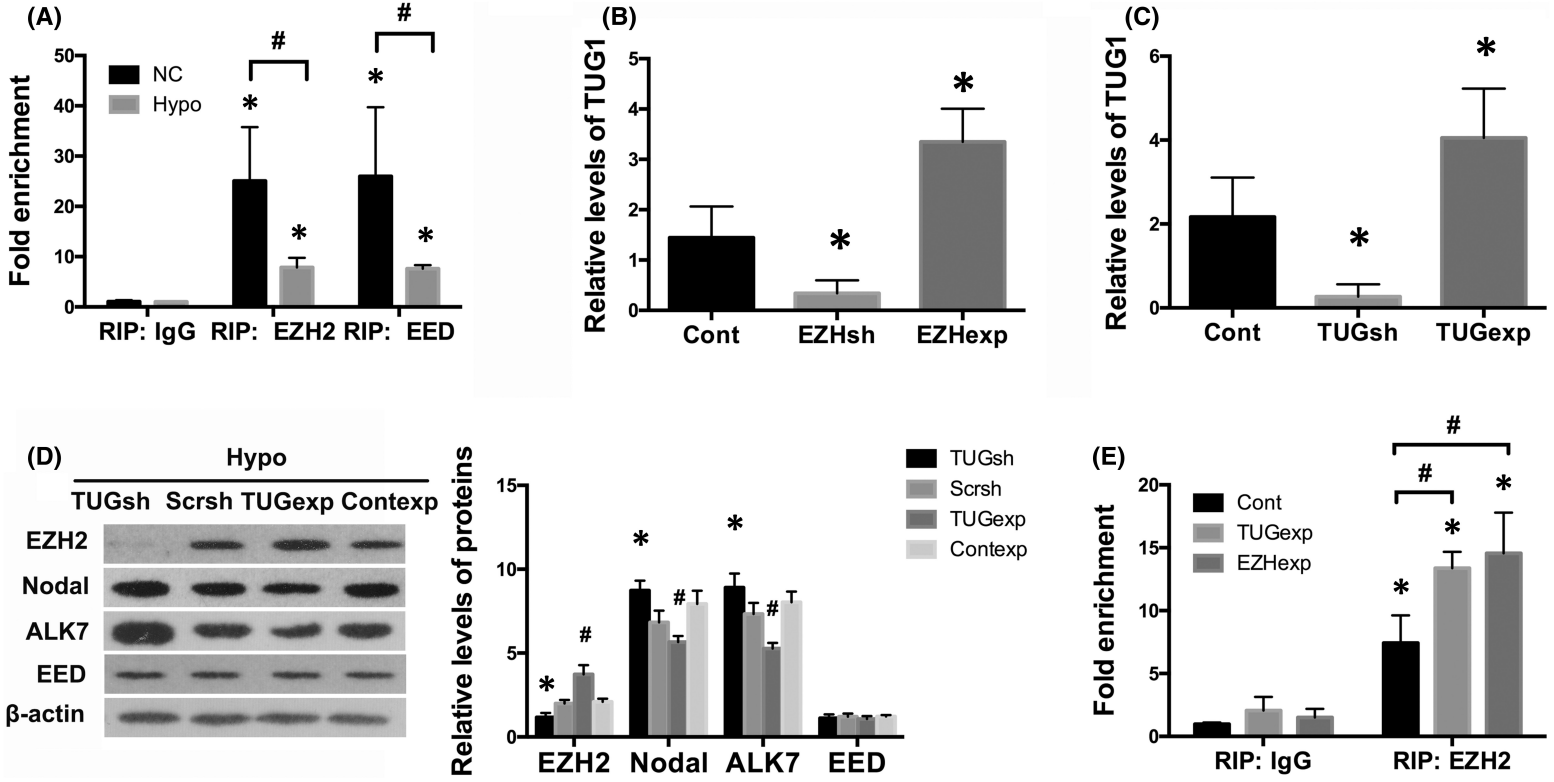
TUG1 was involved in the regulation of Nodal/ALK7 signalling in trophoblast through binding to PRC2. (A) RIP analysis was performed in HTR8/SVneo cells under normal or hypoxic conditions. Real‐time PCR was used to determine the RIP signals. Values denote the mean ± SD; *p < 0.01 versus IgG, ^#^ *p* < 0.05 versus NC. (B) Real‐time PCR showed the impacts of EZH2 gene silencing or overexpression on levels of TUG1 in HTR8/SVneo cells under hypoxic conditions for 24 h. Values denote the mean ± SD; *p < 0.01 versus Hypoxia+ Control vector. (C) Real‐time PCR showed the impacts of TUG1 gene silencing or overexpression on levels of TUG1 in HTR8/SVneo cells in hypoxia for 24 h. Values denote the mean ± SD; *p < 0.01 versus Hypoxia+ Control vector. (D) Western blot analysis showed the impacts of transfection with TUG1 shRNAs or expressing vectors plus hypoxia on protein levels of EZH2, EED, Nodal and ALK7. Values denote the mean ± SD; *p < 0.05 versus Hypoxia+ Scramble shRNA, ^#^ *p* < 0.01 versus Hypoxia+ Control expressing vector. (E) RIP‐qPCR demonstrated differential enrichments of TUG1 in hypoxic HTR8/SVneo cells where EZH2 or TUG1 was overexpressed. Values denote the mean ± SD; *p < 0.01 versus IgG, ^#^ *p* < 0.05 versus Hypoxia+ Control vector

To investigate the interactions between TUG1 and PRC2, we performed RIP in HTR8/SVneo cells and found by real‐time PCR that under ambient air conditions, TUG1 bound to EZH2 and EED in trophoblasts, as demonstrated by a marked amplification of TUG1 in samples using anti‐EZH2 or EED antibodies as compared to IgG (*p* < 0.01; Figure 4A), showing a close interplay between TUG1 and PRC2. In contrast, TUG1 enrichment was significantly reduced under hypoxic conditions (*p* < 0.05; Figure 4A), suggesting that TUG1/PRC2 interaction was partly damaged in hypoxic trophoblasts.

**FIGURE 5:**
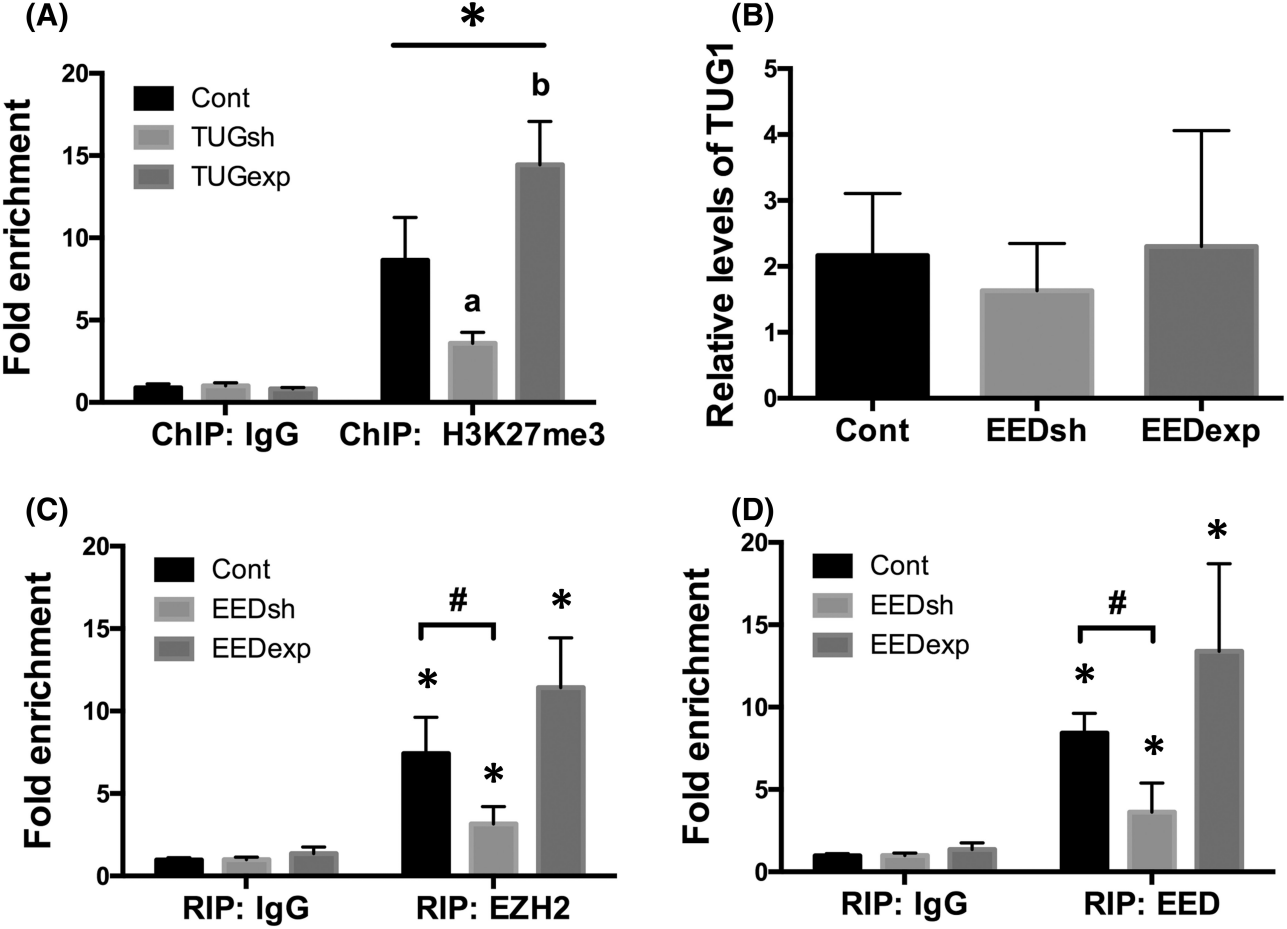
Disruption of TUG1/PRC2 interactions was involved in the regulation of Nodal/ALK7 signalling through decreasing H3K27me3 levels at Nodal gene. (A) ChIP‐qPCR analysis showed differential ChIP signals at the promoter of Nodal gene in HTR8/SVneo cells where TUG1 was prohibited by shRNAs or overexpressed under hypoxic conditions. Values denote the mean ± SD; *p < 0.01 versus IgG, ^a^ *p* <0.05 versus Hypoxia+ Control vector, ^b^ *p* <0.01 versus Hypoxia+ Control vector. (B) Real‐time PCR illustrated the levels of TUG1 after EED gene knockdown or overexpression in HTR8/SVneo cells under hypoxic conditions. Values denote the mean ± SD; *p* > 0.05 versus Hypoxia+ Control vector. RIP‐qPCR analysis demonstrated the mutual binding affinity of TUG1 to EZH2 (C) or EED (D) in HTR8/SVneo cells transfected with EED shRNAs or expressing vectors under hypoxic conditions for 24 h. Values denote the mean ± SD; *p < 0.01 versus IgG, ^#^ *p* < 0.05 versus Hypoxia+ Control vector

Next, we examined TUG1 expression in HTR8/SVneo cells transfected with different vectors and treated with hypoxia for 24 h. Our results revealed by real‐time PCR that EZH2 gene silencing under hypoxic conditions further downregulated TUG1 expression (*p <* 0.01; Figure 4B). EZH2 gene overexpression, on the contrary, fostered TUG1 levels (*p* < 0.01; Figure 4B). TUG1 shRNAs transfection plus hypoxia further decreased TUG1 levels (*p* < 0.01; Figure 4C), together with a greater degree of reduction in EZH2 expression (*p* < 0.05; Figure 4D), whereas overexpression of TUG1 not only led to upregulated TUG1 expression (*p* < 0.01; Figure 4C), but also gave rise to EZH2 expression (*p* < 0.01; Figure 4D), implying a potential positive feedback between TUG1 and EZH2. Moreover, overexpression of EZH2 or TUG1 also increased their mutual binding in hypoxia, as evidenced by increased enrichment of TUG1 in HTR8/SVneo cells where EZH2 (*p* < 0.01) or TUG1 (*p* < 0.05) was overexpressed, in comparison with controls (Figure 4E). Meanwhile, TUG1 shRNA transduction in hypoxia caused a further loss of H3K27me3 deposition at Nodal promoter (*p* < 0.05; Figure 5A), which was correlated with a further increase in both Nodal (*p* < 0.05) and ALK7 (*p* < 0.05) expression under hypoxic conditions (Figure 4D), in contrast, TUG1 overexpression led to increased H3K27me3 mark at promoter region of Nodal gene (*p* < 0.01; Figure 5A), associated with a decrease in both Nodal (*p* < 0.01) and ALK7 (*p* < 0.01) expression (Figure 4D).

Notably, EED gene knockdown or overexpression under hypoxic conditions for 24 h did not significantly affect TUG1 levels (*p* > 0.05; Figure 5B) or EZH2 expression (*p* > 0.05; Figure 3C), compared with cells transfected with control vectors. However, EED knockdown plus hypoxia treatment did decrease the binding affinity of TUG1 to PRC2, as TUG1 enrichment was reduced using either anti‐EZH2 (*p* < 0.05; Figure 5C) or ‐EED (*p* < 0.01; Figure 5D) antibodies in HTR8/SVneo cells, compared with cells transfected with control vectors. EED overexpression only led to a slight but not remarkable increase in mutual binding between TUG1 and PRC2 (*p* > 0.05; Figure 5C,D). Moreover, interference with the expression of TUG1 (*p* > 0.05; Figure 4D) or EZH2 (*p* > 0.05; Figure 3B) did not significantly affect EED downregulation resulted from hypoxia. These aforementioned results suggested that EED might play as a critical mediator for TUG1 and EZH2 binding, and that disrupted TUG1/PRC2 interaction was responsible for ectopic activation of downstream Nodal/ALK7 signalling in injured trophoblasts under hypoxic conditions.

### 3.4 Disruption of TUG1/PRC2 interaction impaired trophoblast invasion via reducing MMP2 expression in a Nodal/ALK signalling‐dependent manner

**FIGURE 6:**
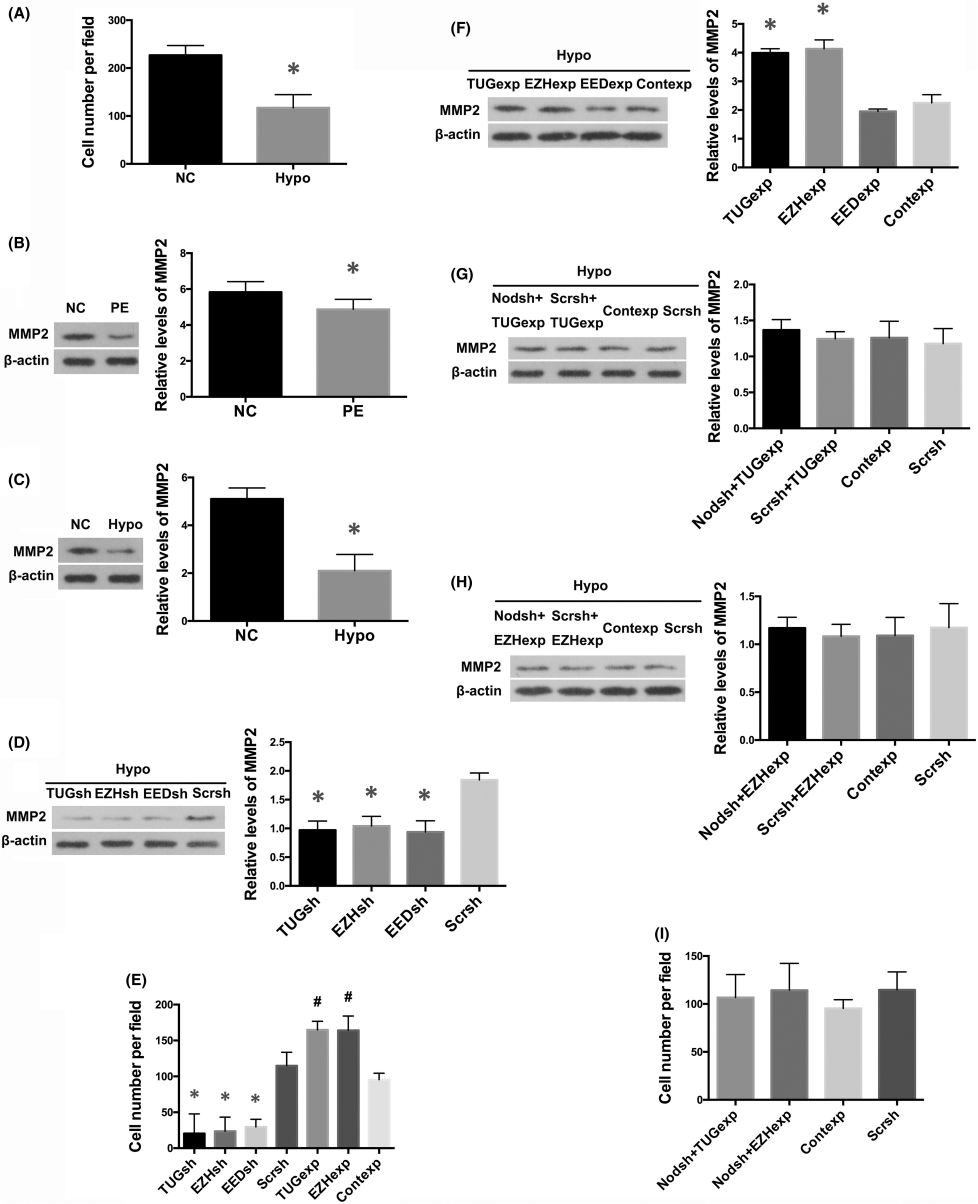
Disruption of TUG1/PRC2 interactions impaired trophoblast invasion via reducing MMP2 expression. (A) Transwell invasion assay showed decreased cell invasiveness of HTR8/SVneo cells after treatment of hypoxia for 24 h, compared with normal trophoblasts. Values denote the mean ± SD; *p < 0.05 versus NC. Western blot analysis showed that the expression of MMP2 was downregulated in preeclamptic placentas (B), as well as in HTR8/SVneo cells in hypoxia for 24 h (C), in comparison with their controls, respectively. Values denote the mean ± SD; *p < 0.01 versus NC. (D) Western blot analysis indicated the protein levels of MMP2 in HTR8/SVneo cells transfected with different shRNAs targeting TUG1, EZH2 or EED under hypoxic conditions for 24 h. Values denote the mean ± SD; *p < 0.01 versus Hypoxia+ Scramble shRNA. (E) Transwell invasion assay indicated the alteration of invasion property of HTR8/SVneo cells transfected with different shRNAs or expressing vectors in hypoxia for 24 h. Values denote the mean ± SD; *p < 0.01 versus Hypoxia+ Scramble shRNAs, ^#^ *p* < 0.05 versus Hypoxia+ Control expressing vector. (F) Western blot analysis illustrated the effects of overexpression of TUG1, EZH2 or EED on MMP2 protein levels in HTR8/SVneo cells treated with hypoxia for 24 h. Values denote the mean ± SD; *p < 0.01 versus Hypoxia+ Control expressing vector. (G) Western blot analysis illustrated the effects of dual transfection of Nodal shRNAs and TUG1 expressing vectors on MMP2 protein levels in HTR8/SVneo cells under hypoxic conditions for 24 h. Values denote the mean ± SD; *p* > 0.05 versus Hypoxia+ Scramble shRNA, *p* > 0.05 versus Hypoxia+ Control expressing vector. (H) Western blot analysis showed the impacts of dual transfection of Nodal shRNAs and EZH2 expressing vectors on MMP2 protein levels in HTR8/SVneo cells under hypoxic conditions for 24 h. Values denote the mean ± SD; *p* > 0.05 versus Hypoxia+ Scramble shRNA, *p* > 0.05 versus Hypoxia+ Control expressing vector. (I) Transwell invasion assay showed little impacts of dual transfection of Nodal shRNAs and TUG1 or EZH2 expressing vectors on cell invasiveness of HTR8/SVneo cells under hypoxic conditions for 24 h. Values denote the mean ± SD; *p* > 0.05 versus Hypoxia+ Scramble shRNA, *p* > 0.05 versus Hypoxia+ Control expressing vector

Impaired trophoblastic invasion and spiral artery remodelling are widely considered as hallmarks of PE.^3^, ^4^ In the present study, transwell assay showed that the invasion capabilities of HTR8/SVneo cells were diminished under hypoxic conditions (*p* < 0.05; Figure 6A). MMPs play an important role in endometrial tissue remodelling during pregnancy, and are highly related to trophoblast cell invasiveness.^6^ In the present study, we showed that MMP2 levels were reduced in severe PE placentas (*p* < 0.01; Figure 6B), as well as in HTR8/SVneo cells under hypoxic conditions (*p* < 0.01; Figure 6C), compared with their controls, respectively.

Next, we examined the expression of MMP2 and cellular invasion capabilities in HTR8/SVneo cells transfected with different vectors and treated with hypoxia for 24 h. Results indicated that gene knockdown of TUG1, EZH2 or EED under hypoxic conditions caused further reduction in expression of MMP2 (*p* < 0.01; Figure 6D), with further reduced cell invasion (*p* < 0.01; Figure 6E), compared with cells transfected with control vectors. TUG1 or EZH2 overexpression partially rescued the downregulation of MMP2 (*p* < 0.01; Figure 6F), and invasion capabilities caused by hypoxia (*p* < 0.05; Figure 6E). And EED overexpression did not significantly altered MMP2 reduction by hypoxia (*p* > 0.05; Figure 6F).

Furthermore, we transfected HTR8/SVneo cells where TUG1 or EZH2 was overexpressed with Nodal shRNAs and treated with hypoxia. Results revealed that in both groups of cells, neither the aberrant expression of MMP2 (*p* > 0.05; Figure 6G,H), nor the reduced invasion caused by hypoxia was significantly affected (*p* > 0.05; Figure 6I). These results indicated that disruption of TUG1/PRC2 interaction damaged trophoblast invasion capabilities via reducing MMP2 expression, at least in part, in a Nodal/ALK7 signalling‐dependent manner under hypoxic conditions.

## 4 DISCUSSION

Inadequate trophoblastic invasion has been widely considered as one of hallmarks of PE.^3^, ^4^ Increasing evidence has indicated that aberrant expression of lncRNAs are involved in preeclamptic placenta.^12^ TUG1 is a 7598‐bp lncRNA gene located on human chromosome 22q12.2, characterized as an oncogene closely correlated with cell proliferation, invasion and migration.^18^ In the present study, we showed remarkable reduction of TUG1 expression in severe preeclamptic placenta and in HTR8/SVneo cells under hypoxic conditions, along with reduced expression of MMP2, as well as impaired trophoblastic invasion, which were worsened by TUG1 gene knockdown but partially rescued by TUG1 overexpression. MMPs are zinc‐dependent proteases that contribute to tissue remodelling via its capability to breakdown of extra‐cellular matrix (ECM),^39^, ^40^, ^41^ and thus, ensure adequate cytotrophoblast invasion of spiral arteries and placental remodelling maintaining the blood supply to developing fetus.^42^, ^43^ Abnormal expression of a series of MMPs, including MMP2, MMP3, MMP9 and MMP12, together with dysregulated inflammation and oxidative factors such as IL‐6, IL‐35, tumour necrosis factor‐α and iNOS, may cause excessive collagen deposition, and in turn lead to decreased ECM remodelling, shallow trophoblast invasion and poor spiral arteries remodelling.^40^, ^41^, ^44^, ^45^, ^46^, ^47^ In the present study, our findings provided evidence that TUG1 dysregulation might play a key role during the pathogenesis of PE, in close correlation with ectopic expression of MMP2, and thus, impaired trophoblast invasiveness.

Reportedly, TUG1 is predominantly located in the nucleus and may be involved in gene regulation at the transcriptional level.^48^ Here, in the present study, we showed TUG1 bound to both EZH2 and EED in normal HTR8/SVneo cells, suggestive of a close interaction between TUG1 and PRC2 in trophoblasts in vitro. And this binding affinity was enhanced by TUG1 or EZH2 overexpression. PRC2 is a large multimeric complex, compromising of four core components (in humans): EZH2, EED, SUZ12 and RbAp46/48, which post‐translationally modifies histone tails and is considered to cooperate in transcriptional repression of target genes by altering local, higher‐order chromatin structure.^26^, ^27^, ^28^, ^49^, ^50^ Mutants of mammalian PRC2 or of its substrates contribute to cancers and other pathological processes including diabetes and developmental diseases,^51^ during which lncRNAs have been recognized as important participants.^24^, ^52^ In cancer cells, TUG1 could modulate tumour‐related genetic loci by recruiting and binding to PRC2 protein complexes and promote metastasis by affecting epithelial–mesenchymal transition.^48^, ^53^, ^54^ In trophoblast cells, it was reported that EZH2 was involved in lncRNA HOTAIR‐and UCA1‐induced inhibition of cell proliferation, migration and invasion.^55^, ^56^ In the present study, we showed that jeopardized TUG1/PRC2 interaction by hypoxia treatment resulted in reduced expression of MMP2, as well as decreased trophoblast invasion; and a further disruption of TUG1/PRC2 binding by genetic knockdown of TUG1, EZH2 or EED under hypoxia showed a greater inhibitory effect on expression of MMP2, as well as cell invasion, whereas strengthening of this interplay by overexpression of TUG1 or EZH2 displayed a reversal effect and amelioration of reduced cell invasiveness resulted from hypoxia. Our observations further implied a potentially key role of TUG1 cooperating with PRC2 by affecting trophoblast function and invasion in the setting of placental dysfunction in hypoxia.

According to the literature, PRC2 is the only enzymatic activity found thus far that is responsible for di‐ and trimethylation of histone H3 at lysine 27 (H3K27me2/3) via its enzymatic subunit EZH2.^52^ H3K27 is one of the best‐studied histone modification sites and H3K27me3 is generally considered as a repressive mark of specific genes.^6^, ^25^, ^51^ In the present study, we found that H3K27me3 levels were sharply downregulated in the PE placenta and hypoxia‐treated HTR8/SVneo cells, along with dysregulated PRC2 and TUG1 expression, as well as impaired trophoblastic invasion, suggesting there might exist a epigenetic mechanism. Indeed, we showed that levels of H3K27me3 mark were also declined at the promoter of Nodal gene; and simultaneously, Nodal protein and its downstream receptor ALK7 were elevated in hypoxic trophoblasts. These observations were prohibited by enhanced TUG1/PRC2 interaction by overexpression of TUG1 or EZH2 under hypoxic conditions. Nodal is a member of the transforming growth factor superfamily,^57^ highly expressed in invasive cancers including pancreatic, endometrial and breast cancer and promotes a highly invasive phenotype.^58^ In human cancer cells, accumulation of EZH2 and H3K27me3 at the epigenetic regulatory element were diminished. Also, pharmacological inhibition of PRC2 led to reactivation of Nodal expression in normal mouse embryonic fibroblasts.^59^ Previous studies have illustrated that Nodal regulated trophoblast differentiation and placental development.^60^ Repressive H3K27me3 mark at the promoter of Nodal gene was regulated during embryonic differentiation in mouse ESCs.^36^ Our findings showed for the first time, to our knowledge, that Nodal was epigenetically dysregulated by PRC2 in hypoxic trophoblasts, during which TUG1 participation was potentially indispensable. Studies have demonstrated excessive expression of Nodal was shown to prohibit placental trophoblast invasion during the pathogenesis of PE.^34^, ^61^ Here, in the present study, we further confirmed activation of Nodal/ALK7 signalling abrogated trophoblast invasion under hypoxic conditions. Moreover, we showed interference with Nodal expression in TUG1‐ or EZH2‐overexpressed HTR8/SVneo cells led to limited amelioration effects on MMP2 expression or migration and invasion property. Therefore, it was plausible to infer that hypoxia‐induced disruption of TUG1/PRC2 interplay might jeopardize human trophoblast invasion through reducing MMPs expression in a Nodal/ALK7 signalling‐dependent way.

Although EZH2 possesses intrinsic methyltransferase activity, other components of PRC2 also holds a critical position in maintaining cellular transcriptional memory by silencing gene expression.^25^, ^62^, ^63^ Deletion of EED is correlated with severe effects, such as failure of secondary invasive trophoblast giant cell differentiation, leading to embryonic lethality.^64^, ^65^ Likewise, we showed EED knockdown plus hypoxia treatment decreased the binding affinity of TUG1 to PRC2, compared with hypoxic trophoblasts without transfection, although overexpression of EED did not significantly increase this mutual binding, suggesting an indispensable role of EED in PRC2 function as an epigenetic regulatory element. Additionally, we preliminarily showed that under hypoxic conditions, TUG1 overexpression resulted in upregulation of EZH2 expression, and EZH2 overexpression also led to elevated TUG1 expression, without altering EED expression, suggesting a potentially positive feedback regulation between TUG1 and EZH2 with EED as a key mediator during PRC2 dysfunction in the setting of hypoxic trophoblasts. Of interest, previous studies showed that in breast cancer, HIF1‐α induction upon hypoxia might be a crucial modulator of PRC2 function through selectively suppressing SUZ12 and EED, leading to a functional switch toward EZH2/FoxM1‐dependent induction of cell invasiveness^66^; and reportedly, HIF‐1α was overexpressed in preeclamptic placenta and involved in mutant trophoblast differentiation through transactivating TGF‐β3 promoter activity and elevating endogenous TGF‐β3 expression.^67^ And here we inferred that aberrant alterations of TUG1 and PRC2 observed in the present study might as well be linked to HIF‐1α pathway in trophoblasts under hypoxic conditions or preeclamptic placenta, which, however, still required further in vitro and in vivo investigations.

Taken together, we demonstrated the involvement of TUG1 and PRC2 misregulation during the pathogenesis of PE, and we showed a close interplay between TUG1 and PRC2, disruption of which by hypoxia contributed to aberrant activation of Nodal/ALK7 signalling through epigenetically reducing H3K27me3 mark at Nodal promoter gene; and Nodal/ALK7 signalling activation further resulted in abnormal expression of MMP2, which might cause excessive collagen deposition and thereby diminished trophoblast invasion. Rectification of TUG1/PRC2 interaction markedly prohibited this ectopic downstream signalling pathway, reversed MMP2 expression and rescued trophoblast invasiveness. These observations provided novel evidence for crucial roles of TUG1 and PRC2 in trophoblastic dysfunction, and a further understanding of lncRNAs in biological processes in the pathogenesis of PE.

## 5 CONCLUSION

In summary, our findings disclosed a critical role of dysregulation of lncRNA TUG1 in damaged trophoblast invasion in hypoxia, potentially via its interplay with PRC2 which might thereby epigenetically impact Nodal/ALK7 signalling and its downstream target MMP2, contributing to a novel insight concerning pathways responsible for inadequate trophoblastic invasion in preeclamptic pregnancy. And the findings might shed some light on identifying future therapeutic targets for PE.

## AUTHOR CONTRIBUTIONS

**Mengsi Hu:** Conceptualization (lead); formal analysis (lead); funding acquisition (lead); investigation (lead); methodology (lead); project administration (equal); writing – original draft (lead); writing – review and editing (lead). **Yao Wang:** Conceptualization (supporting); formal analysis (supporting); investigation (supporting); methodology (supporting); project administration (supporting); resources (supporting); software (supporting); supervision (supporting); writing – original draft (supporting); writing – review and editing (supporting). **Yanping Meng:** Conceptualization (supporting); formal analysis (supporting); investigation (supporting); methodology (supporting); project administration (supporting); supervision (supporting); writing – original draft (supporting); writing – review and editing (supporting). **Jinxiu Hu:** Formal analysis (supporting); investigation (supporting); methodology (supporting); resources (supporting); software (supporting). **Jiao Qiao:** Formal analysis (supporting); investigation (supporting); resources (supporting); validation (supporting). **Junhui Zhen:** Investigation (supporting); resources (supporting); supervision (supporting). **Decai Liang:** Formal analysis (supporting); methodology (supporting); resources (supporting). **Minghua Fan:** Conceptualization (equal); data curation (equal); funding acquisition (equal); methodology (equal); project administration (equal); resources (equal); supervision (lead); validation (equal); writing – original draft (equal); writing – review and editing (equal).

## CONFLICT OF INTEREST

The authors declare that there are no conflicts of interest associated with this manuscript.

## Data Availability

All data generated or analysed during this study are included in this article.
